# Educational effectiveness of notebooks for reflecting on lectures in dental pharmacology

**DOI:** 10.1186/s12909-025-08168-6

**Published:** 2025-11-18

**Authors:** Takuma Inouchi, Ryusuke Nakatsuka, Yuka Sasaki, Tadashige Nozaki

**Affiliations:** Department of Pharmacology, Osaka Dental University, 8-1 Kuzuhahanazono-cho, Hirakata-shi, Osaka 573-1121 Japan

**Keywords:** Dental pharmacology, Notebook, Multiple-choice test, Essay test, Memory-creation process, Logical-thinking

## Abstract

**Background:**

In Japanese education system, learning the basic dentistry mainly consists of lectures using PowerPoint without active learning. Presentation-based learning is problematic because students are passive participants, thereby necessitating content review to improve the learning effects. Summarizing lecture contents through notes is considered an effective learning method. However, it remains unclear whether use of notes translates to improved test scores. The present study aimed to evaluate notes taken by students during lectures, analyze the relationships between lecture note evaluation scores and test scores, and present considerations for the use and effects of lecture notes in learning.

**Methods:**

New learners of dental pharmacology were asked to summarize the contents of lectures and practicum sessions on pharmacology in lecture notes. After all lectures and practicum sessions had been completed, multiple-choice objective tests (answer sheets) and essay tests were performed twice each as learning evaluations. Students were allowed to use their lecture notes in the first essay test but not in the second test. The evaluation scores for the lecture notes were recorded, and the relationships with the test scores were analyzed using regression and correlation analyses.

**Results:**

For the multiple-choice objective test, positive relationships were confirmed between the note evaluation scores and the test scores for both the first and second tests. Meanwhile, in the essay test, a positive relationship was only confirmed for the first test when the use of lecture notes was allowed. These findings demonstrate that lecture notes are an effective learning method during the first stage of the “memory-creation process” of learning (study), namely the “encoding” stage. The findings further indicate that, in terms of documenting one’s thoughts, lecture notes functioned as an “external storage device” for memory and that such notes were important for “recalling” learning content.

**Conclusions:**

Active use of lecture notes in multiple-choice objective tests, such as the national examination for Japanese dental practitioners, is an effective learning method. In addition, use of lecture notes during essay tests is an effective method for evaluating students’ ability to compose logical sentences and text by recalling learning content.

## Introduction

Students who seek to become dental practitioner must correctly understand and memorize the content of their studies during lectures and practicum sessions. Naturally, the accumulation of knowledge will be linked with passing the national examination for dental practitioners. Sustained memory of learning content follows the process of “encoding” memory information obtained via classroom study, “retention,” whereby the encoded information is held continuously without memory loss, and “recall,” where the stored memory can be called up as necessary [1]. The national dental practitioner’s examination in Japan demands free and flexible recall of this knowledge, which can be combined into responses to many examination questions. Temporary memory (as “short-term memory”) is insufficient to achieve the knowledge levels demanded of dentists. Thus, students must study daily to firmly fix and retain (i.e., “consolidate”) the study details in their memory. Nevertheless, lectures are the mainstream teaching component in ordinary classrooms, meaning that students become passive learners. This is problematic because lecture details can be only partially and vaguely recalled, resulting in insufficient consolidation of memory for the desired retention and recall [2]. The effects for retaining and recalling lessons are believed to improve through repetition, and thus review-based learning is important.

In such cases, an effective learning method is for the learners themselves to repeatedly review and reflect on the study contents [2]. One review method involves writing lecture notes that can be referred to after class. Summarizing lecture contents in notes can function as encoding of what has been learned. Moreover, it is considered an effective learning method for the processes of memory retention and recall [3–5]. Undoubtedly, textbooks and materials provided by lecturers can aid in encoding the learning content. Nevertheless, studies have shown that note writing and review-based learning are more effective for short-term favorable test results than merely reviewing materials provided by teachers [6]. Writing and use of lecture notes are thought to assist students in arranging and systematizing their knowledge. Previous research has shown that taking notes supports the effective establishment and recall of information from memory. However, note-taking is less helpful for students who are mainly required to answer inference questions [7, 8]. Given that the national examination for dental practitioners places greater demands on knowledge memorization than inferencing, taking notes is an effective approach for dental education students. Moreover, taking notes encourages evaluation of logical thinking, leading to memory recall through review-based learning. However, the ability of students to write and summarize their notes varies, and it is unclear whether good and poor note-taking abilities are reflected in students’ test scores and grades.

Dental pharmacology includes general theory-related content, such as drug metabolisms, action mechanisms, and side effects. Students also require an understanding of theoretical content for pharmacological agents used in various medical fields and situations. At the present time, when drugs are developed daily, the necessary knowledge regarding drugs (medicines) is broad-ranging, and the amount of knowledge that students must gain is enormous. Thus, in the present study, we examined the relationships between lecture notes taken during dental pharmacology courses and consolidation of memory. For this, we evaluated the contents of lecture notes, and conducted analyses for their correlations with written test scores. These analyses facilitate the presentation of considerations regarding the use of lecture notes as an effective method for providing educational guidance.

## Participants and methods

### Descriptor definitions

#### Pretest

Written test undertaken before the term examination on dental pharmacology (Table 1).

**Table 1:** Lecture and test schedule for the dental pharmacology class in the current case

Subject	Term
Orientation	
Lectures and practicum session	54 class units (32 lectures and 22 practicum sessions)
Collecting and scoring the notebooks	
↓	6 days
Return the notebooks	
↓	3 weeks
Pretest	
↓	1 week
Reviewing the pretest	3 class units
↓	2 weeks
Actual test	

#### Actual test

Written test performed at the end of the dental pharmacology term (term examination) (Table 1).

#### Multiple-choice objective test

 Written test with multiple-choice questions to assess students’ factual recall and levels of understanding without subjective judgement. Students were prohibited from using their lecture notes during these tests.

#### Essay test

Written tests on selected topics of dental pharmacology to evaluate students’ reasoning skills based on their factual recall and understanding. Students could use their lecture notes for the pretest essay test but were not allowed to use notes for the actual test.

### Participants

The participants were 122 second-year students of Osaka Dental University, attended the class of dental pharmacology at academic year 2022.

### Lecture schedule

An overview of the dental pharmacology lecture and test schedule at Osaka Dental University is provided in Table 1. In brief, students attended an orientation on the use of notebooks and each student was given a notebook prior to the start of the dental pharmacology lectures. The orientation included a briefing regarding the lecture and test schedule, and students were informed that their notebooks would be evaluated. They were notified that they could use their own evaluated notebooks at pretest but would not be able to use them during the actual test. The students attended 54 class units (70 min per unit), consisting of 32 dental pharmacology lectures and 22 dental pharmacology practicum sessions between 29 September and 23 December 2022. On completion of the lectures and practicum sessions the students’ notebooks were temporarily collected and scored. The notebooks were returned to the students 6 days after collection. The pretest took place 3 weeks after returning the notebooks. Following the pretest, three class units were provided for review. Two weeks later the actual test took place.

### Lecture notes

Notes were taken freely for the contents of the lectures and the practicum session. Students were advised that notes were recommended to summarize the main points of each lecture and yield a better understanding of dental pharmacology. Students were expected to write the notes directly by hand, and attaching or inserting copied text from lecture materials or other students’ notes into one’s notes was prohibited. In principle, the notebooks used for note-taking were prepared beforehand and distributed to the students, and students used the same notebooks throughout the lecture. When multiple notebooks were required, additional notebooks were provided to students according to need. Students were responsible for managing their own notebooks, and were allowed to undertake additional editing of the notebooks until completion of the lectures and practicum sessions.

### Evaluation of lecture notes

After completion of the lectures and the practicum session, the notebooks of each student were temporarily collected, and the notebook contents (written notes) were evaluated by two evaluators. We used an evaluation rubric based on four criteria to evaluate the quality of the lecture notebooks, as shown in Table 2(a). First we assessed whether all the content from the 32 lectures was covered in the notebooks. This criterion indicated whether students could comprehensively understand the lecture content given that these lectures covered all the pharmacology content required for the Japan National Dental Examination. The second criterion focused on whether students used figures and tables in note-taking. Visual aids enhance understanding and facilitate the organization of complex information in dental pharmacology. The third criterion was whether the lecture content was appropriately summarized. Simply copying the contents of the lecture slides into notebooks was considered passive learning. Thus, this criterion was established to evaluate active thinking about the content and determine whether taking notes could contribute to a deeper understanding of the lectures. The final criterion considered students’ ability to identify key points in the lecture content, noting whether important points were emphasized and clearly highlighted. Scores were assigned based on the number of criteria met: 20 points for meeting all four criteria, 10 points for meeting three, 5 points for meeting two, 1 point for meeting one, and 0 points for meeting none of the criteria (Table 2(b)). To ensure fairness and consistency of scoring, two evaluators independently assessed the notebooks. After the two evaluators had assessed the notebooks, the final scores were compared. If the final evaluation differed between the two evaluators, they reviewed and discussed each scoring criterion until a final consensus was reached that gave a definitive evaluation, ensuring fairness and consistency.

**Table 2:** Evaluation rubric for lecture notes

Criteria	Evaluation
(a) Evaluation criteria	
Are all lecture contents covered in the notebooks?	Achieved/Not Achieved
Are figures and tables used in note-taking to enhance understanding and organization of content?	Achieved/Not Achieved
Are the lecture contents appropriately summarized?	Achieved/Not Achieved
Are important points emphasized and clearly highlighted (e.g., underlining, bolding, or color coding)?	Achieved/Not Achieved

Number of Criteria Achieved	**Score**
(b)Scoring rules	
4	20 points
3	10 points
2	5 points
1	1 point
0	0 points

To confirm that all students had presented their notebooks, each notebook was stamped before its return to the student. In the test during which notebook usage was permitted, only notebooks with confirmation stamps were allowed to be taken into the test.

### Written tests

The written tests were performed twice, as a pretest and an actual test. The questions in the pretest and actual test covered the same concepts and were created using the same format, although the content of the two tests differed. The tests encompassed the full range of content from the 54 class units, including 32 dental pharmacology lectures and 22 practicum sessions. The difficulty levels of the two tests were carefully adjusted to ensure they were as similar as possible. Each test procedure included a multiple-choice objective test using answer sheets like as national dental practitioner’s examination and an essay test written in longhand. The pretest was conducted on January 6, 2023, two weeks after completing all 54 class units. For the pretest, a 40-minute multiple-choice objective test was completed, followed by a 10-minute break period, and a 30-minute essay test. For the essay test, the students were allowed to use their lecture notes under the condition that the notebooks containing the notes were stamped to confirm that they had been presented for evaluation. The actual test took place on January 24, 2023, 19 days after pretest. During this period, three class units were held to explain questions in the pretest, but no other special lectures were conducted and students studied independently for the actual test. For the actual test, a test period of 70 min was set. During this time, the students completed both a multiple-choice objective test and an essay test without lecture notes. Each multiple-choice objective and essay test was scored, with 100 points being the perfect score for each test **(**Table 3**)**.

**Table 3:** Lecture note evaluation scores and pretest and actual test scores for the multiple-choice objective test and the essay test

	Note evaluation scores (maximum: 20 points)	Multiple-choice objective test scores (maximum: 100 points)		Essay test scores (maximum: 100 points)	
		Pretest	Actual Test	Pretest	Actual Test
Mean	8.1	74.0	76.6	56.5	39.9
Minimum	0	23	42	10	0
Maximum	20	99	98	100	100
Median	5.0	74	78	51	36
Standard deviation	6.04	15.53	11.18	22.13	29.39
Standard error	0.55	1.41	1.01	2.00	2.66

### Tabulation and analysis of results

The scores for the tests and the points allocated to the lecture notes were summed. Graphs were created and analyzed using GraphPad Prism software (GraphPad Software, USA). For each comparison, linear regression analysis was performed when evaluating the relationship between note evaluation scores and test scores, and the contribution rate (*R*^2^) was calculated. The contribution rate (*R*^2^) indicates the fraction in total variation of the dependent variable that is explained by the regression. For the comparisons between multiple-choice objective tests and essay tests, correlation analysis was performed, and Pearson correlation coefficients (*r*) were calculated.

## Results

### Relationship between the lecture note evaluations and the multiple-choice objective tests

**Fig. 1:**
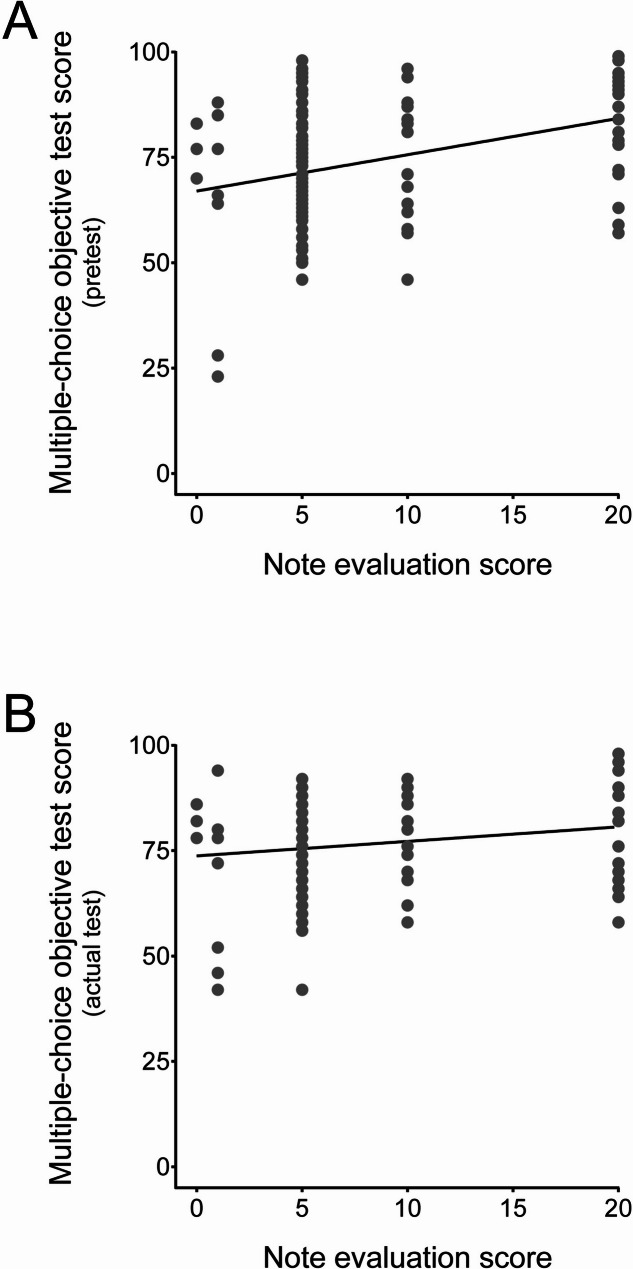
Relationship between the note evaluation scores and the multiple-choice objective test scores. The note evaluation scores (assigned 0, 1, 5, 10, or 20 points) are shown on the X-axis. The multiple-choice objective test scores for the pretest **(A)** and the actual test **(B)** are shown on the Y-axis. Regression lines are indicated in each graph

To investigate the relationship between the note evaluation scores and the multiple-choice objective test scores, graphs were plotted for the pretest **(**Fig. 1A**)** and the actual test **(**Fig. 1B**)**. For both tests, the results confirmed a positive relationship between the note evaluation scores and the test result scores. In the regression analysis, the pretest showed linear form: Y = 0.8628X + 66.99 and contribution rate: *R*^2^ = 0.1126, and the actual test showed linear form: Y = 0.3440X + 73.76 and contribution rate: *R*^2^ = 0.03457.

### Relationship between the lecture note evaluations and the essay tests

**Fig. 2:**
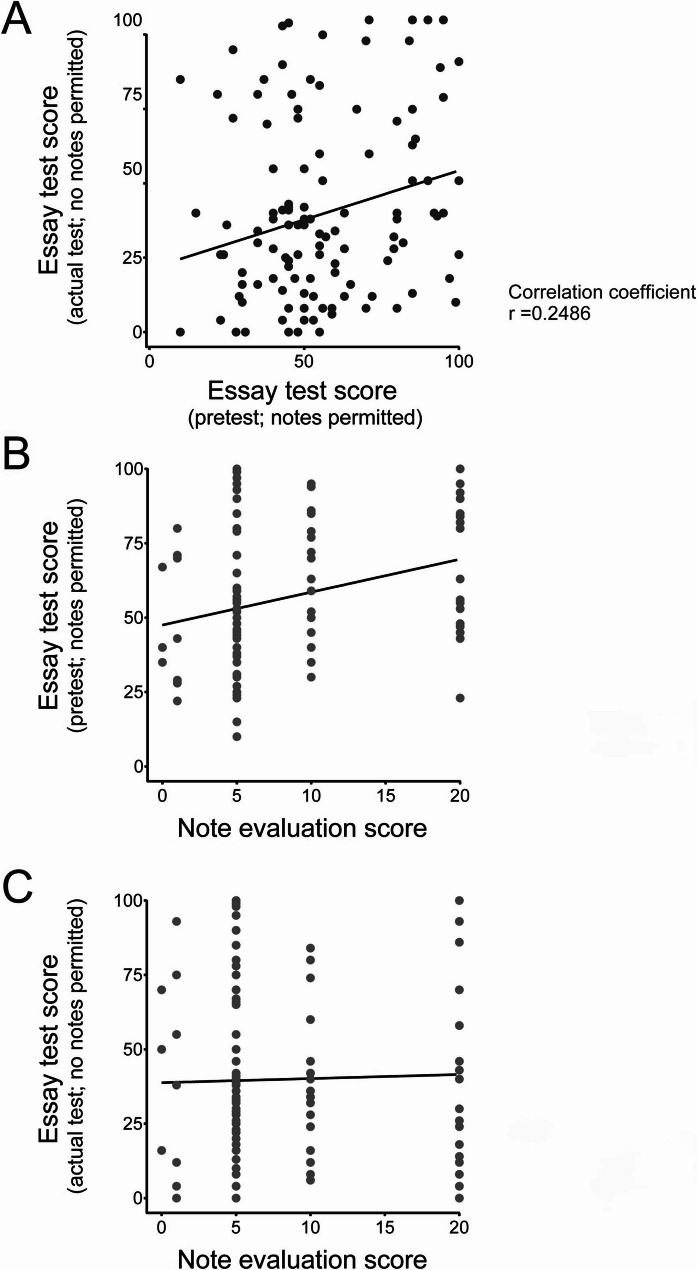
Relationship between the note evaluation scores and the essay test scores. **A** Scatter plot of the essay test scores for the pretest and the actual test. Correlation coefficients (r) are indicated in the graph. **B**,** C** Relationship between the note evaluation scores and the essay test scores. The note evaluation scores (assigned as 0, 1, 5, 10, or 20 points) are shown on the X-axis. The essay test scores for the pretest **B** and the actual test **C** are shown on the Y-axis. Regression lines are indicated in each graph

For the essay tests, the students were allowed to bring in their notebooks and use their lecture notes in the pretest, but not in the actual test. The average scores were lower for the actual test, during which students were not allowed to use their lecture notes **(**Table 3**)**. The results for the pretest and the actual test were plotted, and their correlation analysis were performed **(**Fig. 2A**)**. The correlation analysis showed linear form: Y = 0.3301 X + 21.25 and correlation coefficient: *r* = 0.2486.

To investigate the relationship between the lecture note evaluation scores and the essay test scores, graphs were plotted for the pretest **(**Fig. 2B**)** and the actual test **(**Fig. 2C**)**. The regression analysis between the essay test score on pretest and note evaluation score showed linear form: Y = 1.106X + 47.53 and contribution rate: *R*^2^ = 0.09120. In contrast, the regression analysis between the essay test score on the actual test and note evaluation score showed linear form: Y = 0.1373X + 38.79 and contribution rate: *R*^2^ = 0.0007971. These results suggested that high note evaluation scores are not necessarily linked to high scores in the essay test under the condition where notes were not permitted.

### Relationship between the multiple-choice objective tests and the essay tests

**Fig. 3:**
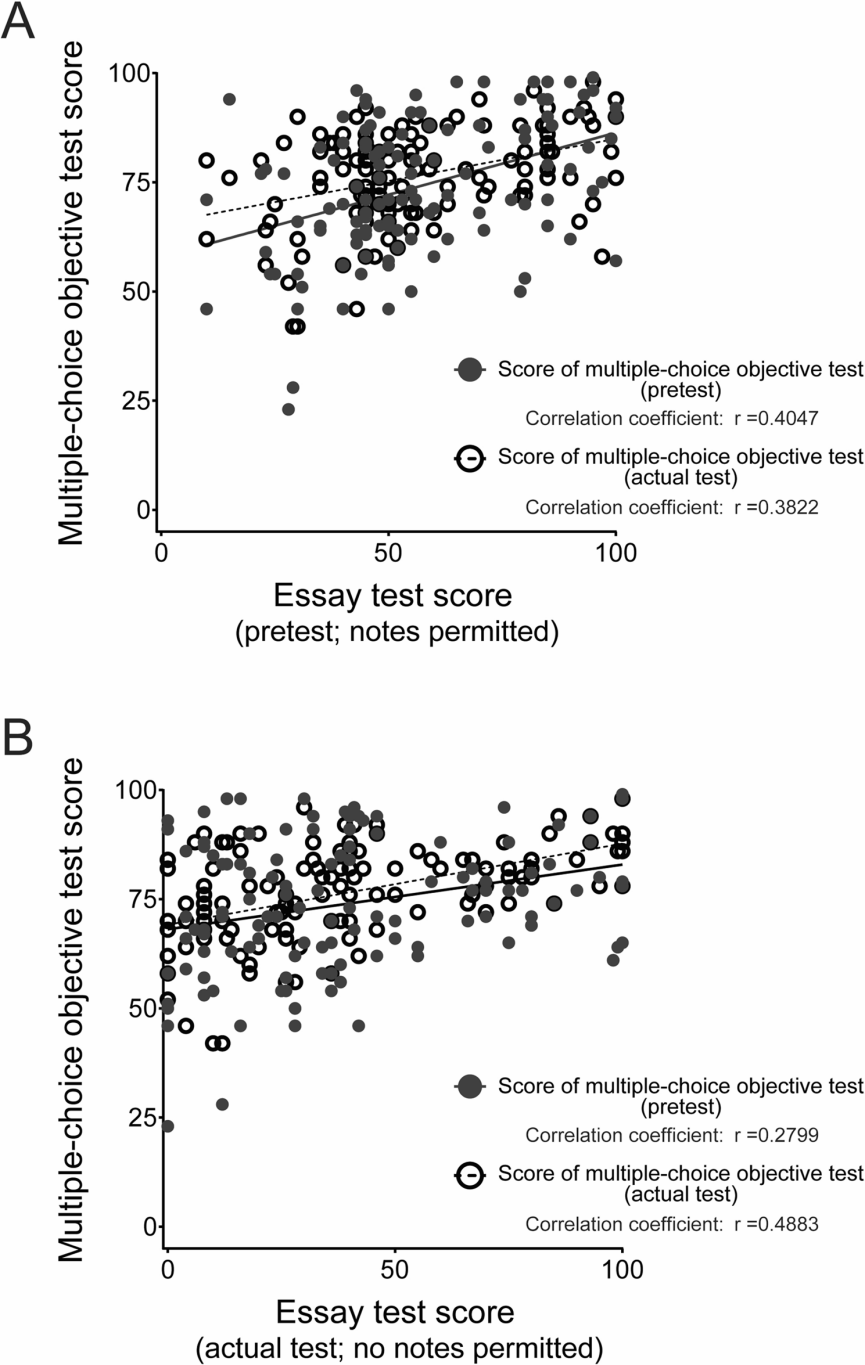
Correlations between the multiple-choice objective tests and the essay tests. The essay test scores for the pretest **(A)** and the actual test **(B)** are shown on the X-axis. The students completed the essay tests with (**A**, pretest; notes permitted) or without (**B**, actual test; no notes permitted) use of their lecture notes. The multiple-choice objective test scores are shown on the Y-axis. Closed and open symbols indicate the multiple-choice objective test scores for the pretest and the actual test, respectively. Correlation coefficients (r) are indicated in each graph

We investigated the correlations between the results of the multiple-choice objective tests and the essay tests (with and without note usage). Graphs were plotted for the pretest essay test (notes permitted) and the pretest and actual test multiple-choice objective tests **(**Fig. 3A**)**, as well as for the actual test essay test (no notes permitted) and the pretest and actual test multiple-choice objective tests **(**Fig. 3B**)**. The correlation analysis between the pretest essay test (notes permitted) and the pretest multiple-choice objective test revealed linear form: Y = 0.2840X + 57.95 and correlation coefficient: *r* = 0.4047. Furthermore, the correlation analysis between the pretest essay test (notes permitted) and the actual test multiple-choice objective test showed linear form: Y = 0.1930X + 65.65 and correlation coefficient: *r* = 0.3822 **(**Fig. 3A**)**. For the actual test essay test (no notes permitted) and the pretest multiple-choice objective test, the correlation analysis showed linear form: Y = 0.1480X + 68.10 and correlation coefficient: *r* = 0.2799. Furthermore, the correlation analysis between the actual test essay test (no notes permitted) and the actual test multiple-choice objective test showed linear form: Y = 0.1857X + 69.14 and correlation coefficient: *r* = 0.4883 **(**Fig. 3B**)**. Based on these results, positive correlations were observed between the essay tests and the multiple-choice objective tests, regardless of whether notes were allowed during the essay tests.

### Relationship between the numbers of notebooks and the test results

**Fig. 4:**
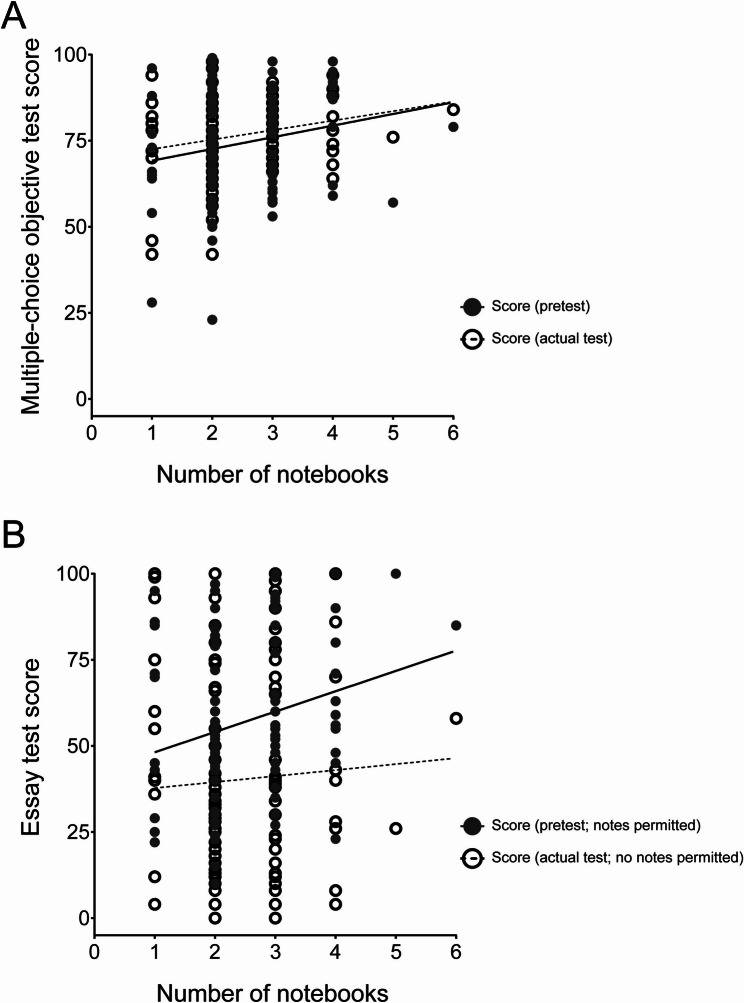
Relationship between the numbers of notebooks and the test results.** A** Scatter plot of the multiple-choice objective test scores and the numbers of notebooks. **B** Scatter plot of the essay test scores and the numbers of notebooks. The numbers of notebooks are shown on the X-axis and the test scores are shown on the Y-axis. Closed and open symbols indicate the essay test scores for the pretest and the actual test, respectively. Regression lines are indicated in each graph

To investigate whether the amount of notes affected the test results, the numbers of notebooks submitted and the results of the multiple-choice objective tests and essay tests were analyzed. For the multiple-choice objective tests, the contribution rates were *R*^2^ = 0.03696 (pretest) and *R*^2^ = 0.04902 (actual test), and regression forms were Y = 3.390X + 65.81 for the pretest, and Y = 2.763X + 69.76 for the actual test. Thus, a weak relationship was indicated for both multiple-choice objective tests (pretest and actual test) **(**Fig. 4A**)**. Moreover, for the essay tests, the contribution rate were *R*^2^ = 0.05523 (pretest) and *R*^2^ = 0.002762 (actual test), and the regression forms were Y = 5.906X + 42.24 for the pretest, and Y = 1.737X + 36.02 for the actual test. Thus, a positive relationship was found only for the pretest essay test, where notebook usage was permitted **(**Fig. 4B**)**.

## Discussion

This analysis of the relationships between the note evaluation scores and the test results revealed that active use of lecture notes in dental pharmacology learning was linked with improved scores in multiple-choice objective tests and essay tests. Positive correlations were also observed between the multiple-choice objective tests and the essay tests. For the notebook evaluations, emphasis was placed on thorough and well-summed content, i.e., whether the information from lectures and textbook contents was efficiently encoded. The study results revealed that as students wrote more superior notes focusing on key points, their scores in the multiple-choice objective tests became higher. Similar results were observed for both the first and second tests. The results suggest a linkage with improved scores in multiple-choice objective tests. While the national examination consists solely of multiple-choice objective questions, logical thinking is necessary to produce the correct answers, and the training and refinement of logical thinking are linked with the cultivation of practical abilities that enable appropriate responses to various issues and problems. It is difficult to achieve logical thinking mastery using only study methods that involve single-minded memorization and the use of test-question booklets and practice tests. The note-writing processes encouraged in the present study are expected to cultivate logical thinking through the efforts made by students to collect and summarize lecture contents in an easy-to-understand way [6].

In the multiple-choice objective tests, students who had created superior notes showed more favorable test scores than those with inferior notes. The lecture notes used in the first test served as a knowledge “memory device,” suggesting that the abilities fostered may be different from those used in the logical construction of written text [9]. The results of the multiple-choice test showed that students with better note-taking skills performed better on the test. This finding corresponds to previous research that indicated that notes are helpful for recalling knowledge but offer less support where students are mainly required to respond to inference questions [7, 8]. Students’ memory retention may be boosted through the process of taking notes as they reflect on the lecture, and students with strong academic skills may be able to take notes more efficiently. In this study, we could not consider students’ intrinsic abilities and basic academic skills that do not depend on note-taking. Further studies are needed to evaluate these issues. For example, students could be divided into groups based on tests administered before the lecture or they could be examined immediately following each class to estimate the knowledge acquired during the class.

In the essay tests undertaken twice in this study, the students were able to use their lecture notes in the pretest but not in the actual test. The pretest essay tests revealed favorable scores for students who had created superior notes and who had systematically summarized the knowledge they had acquired. In contrast, the results of the actual test showed no correlation between the quality of notes and test scores. Previous research mentioned that handwritten essay tests require both “semantic memory” resulting from rote memorization of content and “episodic memory” based on logical thinking [10, 11]. In this study, we did not expect episodic memory to be improved solely through the process of taking notes. However, the expected results were not obtained. Referring to the lecture notes may have stimulated episodic memory, and repeatedly thinking about ways to summarize the lecture content might have led to its further improvement. This hypothesis suggests that reflecting on the lecture by creating lecture notes cultivates logical thinking and leads to improved episodic memory retrieval. Although we did not evaluate the degree of effort made by students to take notes in this study (e.g., the time taken for creation of notes), consideration of effort would elucidate the significance of taking lecture notes in future studies.

Positive correlations were observed between the results for both types of test (multiple-choice objective test and essay test) regardless of the test performance (pretest and/or actual test). Regarding the multiple-choice objective tests and the essay test of pretest, there was a positive correlation between the note superiority/inferiority (by evaluation scores) and the test results. This indicates that higher student grades were linked with greater note-taking ability. Superior notes were also considered to denote a tendency to increase the “volume” (associated quantity) of results related to this enhancement and reinforcement of class (lecture, practicum) content. Nevertheless, without sufficient editing (selective cutting) and good summarization of class content, much of the note-taking would have entailed simple writing down of the content during the class. This necessitates more investigation and verification regarding note quantity and test results/grades. The study also revealed that students with low test scores may not have had sufficient ability to refine their notes, and that students seemingly lacked the motivation to make good notes and use them effectively. Further studies should examine whether providing low-test-result students with the ability to make notes that summarize and refine key points will actually improve their test scores. It is thought that active use of notes to consolidate memory will have positive effects, and it is hoped that inculcating the regular habit of note-taking with skillful summarization in students will help improve their academic ability.

## Conclusions

The findings of this study suggest that when students make notes that summarize and present lecture content in an easy-to-understand way, this helps to consolidate their memory of the learned content. The findings further suggest that note-taking efforts by students may be an effective way to study for multiple-choice objective tests, including the national dental practitioner’s examination. The findings also demonstrate that lecture notes would assist in knowledge-based memory retention. Furthermore, referring to lecture notes could stimulate episodic memory based on logical thinking, as demonstrated by the results achieved when lecture notes were used during the essay test. The combination of essay tests in which notes can be used and multiple-choice objective tests in which notes cannot used may assist in the evaluation of general learning effects.

## Data Availability

Data supporting the conclusions of this study are available from the corresponding author upon request.
